# Circulating Inflammatory Markers May Mediate the Relationship between Healthy Plant-Based Diet and Metabolic Phenotype Obesity in Women: A Cross-Sectional Study

**DOI:** 10.1155/2022/8099382

**Published:** 2022-04-18

**Authors:** Azam Mohamadi, Farideh Shiraseb, Atieh Mirzababaei, Dorsa Hosseininasab, Niloufar Rasaei, Cain C. T. Clark, Khadijeh Mirzaei

**Affiliations:** ^1^Department of Community Nutrition, School of Nutritional Sciences and Dietetics, Tehran University of Medical Sciences (TUMS), Tehran, Iran; ^2^Department of Nutrition, Science and Research Branch, Islamic Azad University, Tehran, Iran; ^3^Centre for Intelligent Healthcare, Coventry University, Coventry, CV1 5FB, UK

## Abstract

**Background:**

It has been posited that both metabolically healthy obesity (MHO) and metabolically unhealthy obesity (MUHO) could be emergent from diet and inflammatory markers. Thus, we sought to investigate the influence of plant-based diet on MHO and MUHO phenotypes mediated by inflammatory markers in overweight and obese women.

**Methods:**

This cross-sectional study was conducted on 289 women aged ≥18 years, with a body mass index (BMI) ≥25 kg/m^2^. Dietary intake was measured using 147 item food frequency questionnaire, as well as anthropometrics and biochemistry panel, in all participants. Metabolic health phenotypes were considered using Karelis score, while plant-based diet indices (PDI) were evaluated based on 18 food groups, where healthy and unhealthy PDI were identified.

**Results:**

Accordingly, 26.9% of women had MHO and 73.1% had MUHO phenotypes. After adjusting for potential confounders, TGF-*β*1 had a significant inverse association with hPDI (*β*: −0.28; 95% CI: 452.99, −85.25; *P*: 0.004). Moreover, we found that women with higher hPDI had lower odds of MUHO (OR: 0.95; 95% CI: 0.39, 2.30; *P*: 0.03). Regarding the mediatory effect of the inflammatory markers, TGF-*β*1 (*P*: 0.73), IL-*β*1 (*P*: 0.14), and MCP1 (*P*: 0.51) played a role in decreasing the odds of MUHO among hPDI tertiles.

**Conclusion:**

There was a significant inverse relationship between adherence to hPDI and MUHO phenotype in overweight and obese Iranian women. This association appeared to be mediated by TGF-*β*1, IL-*β*1, and MCP1.

## 1. Introduction

Obesity remains a leading public health concern all over the world and is associated with incidence of several major chronic diseases such as cardiovascular disease, type 2 diabetes, hypertension, dyslipidemia, and some types of cancer [1]. A systematic review and meta-analysis, in 2019, reported that the prevalence of obesity in Iranian older adults was 21.4% [2]; where there are criteria based on population- and country-specific cut-off point for Iranians to evaluate obesity, including body mass index (BMI) ≥ 30, waist circumference (WC) ≥ 91 cm for women, and ≥89 cm for men, and abdominal obesity, demarcated by increases in subcutaneous, deep subcutaneous, and intra-abdominal visceral adipose tissue [3–5]. In addition, individuals may be classified, metabolically, as having either healthy or unhealthy obesity on the basis of phenotypes.

Indeed, the metabolically healthy obesity (MHO) phenotype has been defined as favorable lipid profile as well as normal or slight changes in insulin sensitivity, while in metabolically unhealthy obesity (MUHO), these criteria are affected abnormally [6]. Some studies have indicated that an intermediate-stage risk of metabolic disorders is evident in individuals with MHO in comparison with MUHO [7, 8].

It has been demonstrated that obesity and inflammation have a strong relationship, such that excessive fat mass can lead to an inflammatory response and increase markers of inflammation, such as transforming growth factor beta 1 (TGF-*β*1), interleukin-*β*1 (IL-*β*1), and monocyte chemoattractant protein-1 (MCP-1). The proinflammatory cytokines in obesity, emergent from the adipose tissue, are produced by infiltrating macrophages, as well as TGF-*β*1 having a role in the regulation of inflammation, immune function, and glucose homeostasis [9–11]. Thus, the adoption of a lifestyle is important to prevent chronic inflammation and other complications that might develop in obesity and chronic disease related to obesity.

According to cohort study, adopting a healthy plant-based diet may reduce the risk of cardiovascular disease in the general population, irrespective of genetic susceptibility [12]. Higher consumption of saturated fatty acid, salt, sugars, excessive alcohol, and low intake of fruit, vegetables, fiber, omega-3, and egg consumption are factors contributing to chronic disease [13, 14]. However, one review study amounts of eggs consumed by adults have no significant influence on systolic and diastolic blood pressure [15]. To reduce the inflammation factors and chronic diseases, higher intake of healthy plant foods (vegetable and fruit), instead of unhealthy foods (refined grains, sweets, and desserts), is useful [16, 17]. A review study shown that nut consumption especially walnut is contributing to weight reduction and control weight by reduction of fat absorption and appetite [18]. In a randomized controlled trial, significant improvement in weight and BMI was observed in the intervention group which had plant-based diet compared to the group with normal care [19] Moreover, Kahleova et al., also observed a significant association between a plant-based diet with changes in body weight and body composition [20]. Empirical research has demonstrated that dietary patterns may be associated with inflammatory mediators and obesity as an inflammatory-related disease [21–24]. For instance, the positive impacts of dietary patterns, including Mediterranean, Dietary Approaches to Stop Hypertension (DASH), and plant-based diet (PBD) on inflammation, obesity, MHO, and MUHO have been elucidated by several studies [25–28]. Moreover, a review article suggested that PBD could reduce some inflammatory markers, such as IL-6 and C-reactive protein (CRP), but not TNF-*α* [26]. This type of dietary pattern has been defined as having high intake of fruits, vegetables, grains, and legumes, all of which could lead to reductions in both inflammation and obesity [29]. However, to our knowledge, the effect of PBD on MHO and MUHO has not yet been examined. Furthermore, dietary inflammatory index, in which the consumption of food items can increase body inflammation such as saturated fatty acid, trans fatty acids, and refined grains, has been shown to be positively associated with fat mass and concentration of MCP-1 [30]. Thus, adherence to a PBD may be associated with an improvement in obesity-related inflammatory profiles and prevention of chronic disease risks. To the best of our knowledge, this is the first study designed to assess the relationship between a healthy plant-based diet and metabolic obesity phenotype, in addition to investigating the mediating role of inflammatory markers (TGF-*β*1, IL-*β*1, MCP1) in overweight and obese Iranian women. Thus, we sought to investigate the influence of plant-based diet on MHO and MUHO phenotypes which are mediated by inflammatory markers in overweight and obese women.

## 2. Methods and Materials

### 2.1. Study Population

This cross-sectional study was conducted using multistage simple random sampling and participants consisted of 289 women recruited from 20 Tehran Health Centers in 2018. Indeed, 20 health centers were randomly selected from all health centers of the Tehran University of medical sciences. Sampling was such that people who referred to Tehran health centers, if they met the inclusion criteria, were randomly selected to enter the study. Inclusion criteria were age ≥18 years old, with a body mass index (BMI) ≥25 kg/m^2^, without history of hypertension, had no intake of alcohol and opiate drugs, not being pregnant, not being in menopause, not having acute or chronic infection, and exclusion criteria were having history of cardiovascular disease, thyroid, cancer, diabetes, liver, and kidney disease, and smoking. In addition, participants who had been following any arbitrary special dietary regimen, as well as those with chronic disease(s) affecting their diet, or if their daily energy intake was <800 kcal or >4200 kcal [31], were excluded. All participants were asked to provide written informed consent prior to participation, and the study was approved by the ethics committee of Tehran University of Medical Sciences (IR.TUMS.VCR.REC.1395.1234). We calculate the study sample prior to the study. The sample size was computed according to the following formula:

(1) $$\begin{array}{c} n=\left.\left[\left(Z1-\alpha +Z1-\beta \right)\times \surd 1-r2\right]/r\right)\left.2+2\right)=289, \end{array}$$

where *β* = 0.95 and *α* = 0.05, then, with 95% confidence and 80% power, and *r* = 0.37.

### 2.2. Anthropometric Measurements

Body composition, including weight, fat, and lean mass, and waist-to-hip ratio was assessed using a bioelectric impedance analyzer (In Body 770 scanner, Korea) [32]. Also, height was measured to the nearest 0.5 cm by nonelastic tape, while BMI was calculated as weight (kg) divided by height (m^2^). WC measurement was performed at the level of the umbilicus after exhalation. According to the World Health Organization (WHO) criteria for classification of weight, BMI ≥25 kg/m^2^ was considered as overweight, and ≥30 kg/m^2^ as obesity [33].

### 2.3. Biochemistry Measurements

Blood samples were drawn after 12 hours of overnight fasting to assess low-density lipoprotein (LDL-C), high-density lipoprotein (HDL-C), triglycerides (TG), homeostatic model assessment (HOMA), C-reactive protein (CRP), TGF-*β*1, IL-*β*1, and MCP-1, using ELISA. The serum was separated and stored at a temperature of −70°C until the analyses were carried.

### 2.4. Metabolic Health Phenotypes

Karelis criterion was used to estimate metabolic health, considering the following: TG ≤ 1.7 mmol/L or use of lipid-lowering drugs, HDL-C ≥1.3 mmol/L, LDL-C ≤2.6 mmol/L, HOMA ≤2.7, and CRP ≤3.0 mg/L [34]. Subsequently, participants were categorized into two groups as metabolically healthy obesity and unhealthy obesity phenotypes [35, 36], where meeting three or more of the preceding components equated to an unhealthy phenotype.

### 2.5. Dietary Intake Measurement

Dietary intake was collected using a 147-item semiquantitative food frequency questionnaire (FFQ), where its validity and reliability were previously affirmed in an Iranian population [37]. The average consumption frequency was considered as over the past year on a daily, weekly, and monthly basis. Household measures were taken into account for portion sizes and then converted to grams [38]. The food composition table (FCT) of the US Department of Agriculture (USDA) was used to evaluate energy and nutrients. The Iranian FCT was considered for local foods that were not present in the USDA FCT. Moreover, total daily energy intake was examined by considering the sum of each food item energy.

### 2.6. Plant-Based Diet Scores

Plant foods were categorized into healthy and unhealthy groups based on epidemiological knowledge concerning the relationship between food items with obesity and inflammation [26]. First, we generated 18 food groups consisting of animal foods (butter/lard, dairy, egg, fish/seafood, meat, and miscellaneous animal-based foods), healthy (whole grains, fruits, vegetables, nuts, legumes, vegetable oils, and tea/coffee), and unhealthy plant foods (fruit juices, SSBs, refined grains, potatoes, and sweets/desserts), in line with nutrient and standard culinary features. Then, daily values and the number of servings for entire foods incorporated in each of the 18 food groups were summed. In this study, we generated an overall plant-based diet index (PDI) based on the algorithm developed by Martinez-Gonzalez et al. [39], and as suggested by Satija et al. two fitted versions of a healthful plant-based diet index (hPDI), and an unhealthful plant-based diet index (uPDI) [40]. The cut points for the tertiles were calculated for each 18 food groups with an assigned score between 1 and 3 for each tertile. To achieve each participant's indices, we summed the 18 food group scores. The observed score ranges for PDI, hPDI, and uPDI, were <51, 51–57, and >57, respectively, across the groups. It is worth mentioning that a higher amount of all indices is indicative of a lower intake of animal foods. Moreover, alcoholic beverages were not included in our indices due to their diverse association with several health outcomes.

### 2.7. Physical Activity Measurement

The international physical activity questionnaire (IPAQ), validated in Iranian women adults, was used to assess physical activity levels [41]. The participants were asked to answer questions such as the time they spent on walking, moderate, and vigorous physical activity during the last week. Then, the time of each physical activity was converted to minutes per week and calculated as metabolic equivalent of task (MET/minutes/week).

### 2.8. Other Covariates Assessments

Demographic characteristics including age, sex, income, marital status, supplements consumption, socioeconomic status, education, and occupation status were collected. In addition, systolic and diastolic blood pressure (SBP and DBP) were evaluated after 15-min rest, using a Mercury sphygmomanometer.

### 2.9. Statistical Analysis

Participants were categorized according to tertiles of PDI and hPDI scores (tertiles 1: < 51, tertiles 2 : 51–57, and tertiles 3 : >57) and uPDI score (tertiles 1 : <45, tertiles 2 : 45–51, and tertiles 3 : >51). Kolmogorov—Smirnov test and histogram were used to determine the normal distribution of the data. All variables with normal distribution were analyzed by parametric tests. One-way analysis of variance (ANOVA) for continuous variables and chi-square analysis for categorical variables were used to compare subject characteristics and dietary intake across tertiles of plant-based diet score and were reported as mean (SD). Analysis of covariance (ANCOVA) was used to examine demographic characteristics, anthropometric measurements, clinical assessments, and dietary intake across tertiles of PDI, hPDI, and uPDI score, adjusting for age, BMI, physical activity, energy intake, occupation status, economic status, supplement consumption, and income. To examine the association between plant-based diet score and MHO and MUHO, binary logistic regression was used, and reported as odds ratio (OR) and 95% confidence interval (CI). Moreover, linear regression was used to examine the association of MHO and MUHO across the tertiles of PDI, hPDI, and uPDI scores and reported as *β* and CI, and adjusted in model 2, including age, BMI, physical activity, energy intake; model 3, with occupation, economic status, supplement consumption, income; and using the Barrett method. Mediation analysis was performed to assess the mediating effects of TGF-*β*1, IL-*β*1, and MCP1 in models 4, 5, and 6, respectively. In the current study, SPSS software version 26 (Chicago—United State) was used for data analysis, and a *P*-value <0.05 was, a priori, considered statistically significant.

## 3. Results

### 3.1. Study Population Characteristics

In total, 289 women, including 65 MHO (26.9%) and 177 MUHO individuals (73.1%), with a mean age and BMI of 36.5 years old and 31.05 kg/m^2^, respectively, were recruited in the present study. The mean (SD) height and weight of participants were 161.26 (5.92) cm and 80.70 (12.24) kg, respectively.

### 3.2. General Characteristics of Participants across the Tertiles of PDI, hPDI, and uPDI Scores

General characteristics of participants, across the tertiles of PDI, hPDI, and uPDI scores in overweight and obese women are shown in Table 1. Participants with a higher score of hPDI were more physically active (*P*=0.001), and those with a higher score of uPDI used less supplements (26.7%, *P*=0.03). Also, individuals with a higher score of uPDI had a lower BMI (*P*=0.02) and WHR (*P*=0.02), after controlling for age, BMI, physical activity, and energy intake. The hs-CRP level was significantly higher across higher scores of PDI after adjusting confounders (*P*=0.02). However, no significant mean differences were found in IL-*β*1, MCP1, and TGF-*β*1 across PDI, hPDI, and uPDI.

### 3.3. Dietary Intake of Participants across the Tertiles of PDI, hPDI, and uPDI Scores

Dietary intake of participants across the tertiles of PDI, hPDI, and uPDI scores in overweight and obese women are presented in Table 2. Participants in the highest tertiles of PDI had higher intake of polyunsaturated fatty acid (PUFA), linoleic acid, linolenic acid, calcium, zinc, vitamin C, folate, total fiber, whole grains, fruits, dairy, and legumes (*P* < 0.001). In addition, higher adherence of hPDI was associated with lower intake of PUFA, linoleic acid, linolenic acid, potassium, iron, magnesium, selenium, vitamin C, folate, total fiber, fruits, vegetables, nuts, legumes, and sea foods (*P* < 0.001). Furthermore, women with the highest score of uPDI consumed higher cholesterol, refined grain, and lower SFA, MUFA, potassium, calcium, magnesium, zinc, vitamin A, *β* carotene, vitamin D, thiamin, vitamin B6, vitamin B12, whole grain, vegetables, dairy, legumes, sea foods, and animal oils (*P* < 0.001) (Table 2). However, other dietary factors across tertiles of PDI, hPDI, and uPDI showed no significant results.

### 3.4. Association of Inflammatory Markers with PDI, hPDI, and uPDI Scores

Table 3 details the association of inflammatory markers, including IL-*β*1 (pg/ml), MCP1 (pg/ml), and TGF-*β*1 (pg/ml), with PDI, hPDI, and uPDI scores in overweight and obese women. In the crude model, none of the inflammatory markers were associated with PDI, hPDI, and uPDI. After adjusting for age, BMI, physical activity, energy intake, occupation, economic status, supplement consumption, income, there was no significant association of IL-*β*1 and MCP1 with PDI, hPDI, and uPDI. However, TGF-*β*1 had a significant inverse association with hPDI (*β*: −0.28; 95%CI: 452.99, −85.25; *P*=0.004), but not with PDI (crude = *β*: −0.04; 95% CI:−182.20, 96.34; *P*=0.54; adjusted = *β*: 0.02; 95% CI: −190.70, 246.09; *P*=0.80) and uPDI (crude = *β*: 0.11; 95% CI: −42.27, 271.78, *P*=0.15; adjusted = *β*: 0.16; 95% CI: 37.57, 384.44; *P*=0.10).

### 3.5. Association of MHO and MUHO Phenotype across the Tertiles of PDI, hPDI, and uPDI Scores

The association of MHO and MUHO phenotypes across the tertiles of PDI, hPDI, and uPDI scores in overweight and obese women are shown in Table 4. There were no significant associations between MUHO phenotypes with the tertiles of PDI, hPDI, and uPDI scores in the crude and adjusted models (*P* > 0.05). In model 2, with adjustment for age, BMI, physical activity, and energy intake, there was a significant *P*-trend for lowering odds from second (OR: 1.83; 95% CI: 0.70, 4.81; *P*: 0.04) to third tertiles of hPDI (OR: 0.93; 95% CI: 0.42, 2.06; *P*: 0.04), compared to the first tertile. Even after further controlling with occupation status, economic status, supplement consumption, and income in the third model, there were significant decreasing odds of MUHO vs. MHO with moving from second (OR: 1.75; 95% CI: 0.62, 4.89; *P*: 0.03) to third tertiles (OR: 0.95; 95% CI: 0.39, 2.30; *P*: 0.03) as compared to the first tertile. This demonstrated that women with a higher score of hPDI had lower odds of MUHO.

### 3.6. Association of MHO and MUHO across the Tertiles of PDI, hPDI, and uPDI Scores Mediated by Inflammatory Markers

The association of MHO and MUHO phenotypes across the tertiles of PDI, hPDI, and uPDI scores mediated by inflammatory markers in overweight and obese women is presented in Table 5. According to Barrett model for assessment of mediation effects, three inflammatory markers, including TGF-*β*1, IL-*β*1, and MCP1, were included in the models of adjustment. A significant *P*-trend of 0.03 for odds of MUHO across hPDI tertiles was attenuated in the model adjusted with inflammatory markers; TGF-*β*1 (*P*-trend: 0.73, *P*-value: 0.89), IL-*β*1 (*P*-trend: 0.14, *P*-value: 0.17), and MCP1 (*P*-trend: 0.51, *P*-value: 0.60). A nonsignificant *P*-trend for odds of MUHO across PDI tertiles remained in the model adjusted with inflammatory markers, highlighting that TGF-*β*1 (*P*-trend: 0.15, *P*-value: 0.32), IL-*β*1 (*P*-trend: 0.30, *P*-value: 0.50), and MCP1 (*P*-trend: 0.56, *P*-value: 0.63) possessed limited mediating ability, with increasing odds of MUHO among PDI tertiles. *P*-trend for odds of MUHO across uPDI tertiles remained nonsignificant in the model adjusted with inflammatory markers. Moreover, scores of uPDI increased nonsignificantly from the second to third tertiles, after controlling for MCP-1 (*P*-trend: 0.63, *P*-value: 0.85), TGF-*β*1 (*P*-trend: 0.38, *P*-value: 0.68), and IL-*β*1 (*P*-trend: 0.86, *P*-value: 0.74). Thereby suggesting limited mediating effectiveness of the three inflammatory markers.

## 4. Discussion

In this cross-sectional study, we presented, for the first time, the relationship between a healthy plant-based diet and metabolic obesity phenotype, in addition to investigating the mediating role of inflammatory markers (TGF-*β*1, IL-*β*1, MCP1), in overweight and obese women.

In the present study, hPDI and uPDI had no significant association with MHO and MUHO in the crude model, but after adjusting confounders, we noticed that women with higher adherence to hPDI, had a lower risk of MUHO phenotype. Kouvari et al. demonstrated that higher adherence to a plant-based diet was associated with a greater probability of long-term maintenance of a healthy metabolic state. In addition, the healthful or unhealthful food choices within this pattern appeared to strongly predict cardiometabolic condition in women [42].

In our study, we investigated the potential mediatory role of TGF-*β*1 in the association between healthy plant-based diet and metabolic phenotype obesity. Indeed, after adjusting for potential confounders, TGF-*β*1 had a significant inverse association with hPDI, in addition, the hs-CRP level was significantly higher across scores of PDI after adjusting confounders. The findings of Pourreza et al.'s study also support our findings, where the authors asserted that PDI was significantly associated with TGF-*β*1, in addition to a strong inverse association between adherence to hPDI and hs-CRP, and a significant positive association between uPDI and hs-CRP [43]. In contemporary work, in a cross-sectional study containing 240 middle-aged women, hPDI was significantly associated with reduced inflammatory biomarkers compared to uPDI [16]. The previous studies were shown consumption of healthy plant foods (vegetable and fruit), instead of unhealthy foods (refined grains, sweets, and desserts), can reduce the inflammation factors and chronic diseases [16, 17].

In another study, Kim et al. indicated that individuals in the highest quintile of uPDI had greater odds of metabolic syndrome (MetS) than those in the lowest quintile. Higher uPDI score was associated with higher odds of hypertriglyceridemia in men, and abdominal obesity, high fasting glucose, and hypertriglyceridemia in women, which was consistent with our study [44]. Moreover, in the present study, higher scores of uPDI were significantly associated with higher BMI, WHR, body fat percentage, TG, and LDL-C. One randomized controlled trial, showed significant reduction in weight and BMI in the intervention group which consumed plant-based diet compare to the group with normal care [19]. Also, Kahleova et al. observed a significant association between a plant-based diet and body weight reduction and body composition changes [20]. While in Kim et al. the positive associations between unhealthy plant-based diets and the components of MetS, such as abdominal obesity, high fasting glucose, and hypertriglyceridemia, were observed only in women. Moreover, the median score of uPDI was moderately higher in women than men in the highest quintiles of uPDI, and the authors posited that women may have consumed more unhealthy plant foods, such as refined grains (e.g., white rice or noodles), than men [44].

There are various mechanisms through which plant-based diets may be associated with obesity and inflammation. Indeed, plant-based diets may reduce body fat through decreased caloric intake and increased energy expenditure due to increased thermogenesis. Polyphenols and unsaturated fatty acids can affect the liver, muscle, and adipose tissue, to help upregulate the expression of peroxisome proliferator-activated receptor (PPAR), which augments oxidation leading to a reduced circulating pool of free fatty acids (FFAs), thereby reducing the accessibility of FFA for adipose tissue uptake and hypertrophy. Reduced use of saturated fats, which are commonly derived from animal-based foods, may also contribute to better insulin sensitivity [45]. Plant-based foods are a major source of phytochemicals, which can act as ligands, substrates, inhibitors, and cofactors for multiple enzymes [46]. The use of phytochemicals, particularly polyphenols, which are available in various plant foods (e.g., berries, grapes, onions, apples, cacao, green tea, soy, and whole grains), are associated with decreased mortality and chronic disease risk [47–50]. Adopting a plant-based diet has been shown to reduce cardiovascular disease risk in the general population, regardless of genetic susceptibility [12]. Based on a review study, walnut consumption has been associated with weight loss and weight control by reducing fat absorption and appetite [18]. Polyphenols are hydroxylated bioactive compounds that can also affect body fat, and an inverse association between polyphenol utilization and body weight has been reported [51, 52]. The food compound of an unhealthy plant-based diet may have higher intakes of undesirable nutrients and lower intakes of micronutrients and antioxidants, which can unfavorably affect metabolic syndrome and its (co)factors. Moreover, a high intake of added sugar from unhealthy plant foods would negatively affect lipid metabolism, glucose control, and weight gain [53], while decreased dietary fiber may impact glycemic control, insulin sensitivity, and lead to increases inflammation. Indeed, these effects could be related to reduced inflammation and oxidative stress [54, 55]. Hence, plant-based diets may provide advantages in the inhibition of chronic disease beyond decreased fat mass. Systemic concentrations of proinflammatory mediators are known to be higher in obese (BMI 30 kg/m^2^) vs. normal-weight persons [56, 57]; indeed, the elevated abdominal fat mass is related to a chronic increase of the circulating concentrations of inflammatory mediators containing multiple acute-phase inflammatory proteins including CRP [58, 59]. It should be noted that the liver and the lymphoid organs are generally the main production sites of these inflammatory mediators, but in obesity, adipose tissue becomes the main producer, resulting in chronic and permanent local and systemic inflammatory [60].

The present study possesses numerous strengths and limitations. First, to the best of our knowledge, this is the first study to have evaluated the association between a healthy plant-based diet and metabolic phenotype obesity and also investigated the potential mediating role of inflammatory markers (TGF-*β*1, IL-*β*1, and MCP1) in overweight and obese Iranian women. Second, another strength of this study is the recruitment of a large sample of obese and overweight individuals. In addition, dietary intake was assessed using a locally validated questionnaire, the FFQ which was completed via interview with an experienced dietitian to minimize measurement errors. Nevertheless, despite these strengths, we must acknowledge some limitations in the present study. First, the cross-sectional nature of this study limited the ability to suggest a causal relationship between a healthy plant-based diet and metabolic phenotype obesity. Second, small errors may be present in the dietary assessment, mostly due to misremembering the data and misclassification errors. Third, because our study only included women, the results are not generalizable to men, although clearly this was not the aim of the study.

## 5. Conclusion

In conclusion, a higher hPDI score was associated with a lower MUHO phenotype in overweight and obese Iranian women, which could be mediated by TGF-*β*1, IL-*β*1, and MCP1. Based on these data, consumption of a plant-based diet containing unrefined and whole plant-foods may have beneficial health effects. It is vital to consider the quality of plant foods consumed in the general population for the improvement of health outcomes.

## Figures and Tables

**Table 1:** General characteristics of participants across the tertiles of PDI, hPDI, and uPDI scores in overweight and obese women (*n* = 289).

	Total (n = 289)	PDI				hPDI				uPDI			
		*T*1 ≤51 (n = 111)	*T*2 51–57 (n = 90)	*T*3 ≥57 (n = 88)	*P*-value	*T*1 ≤51 (n = 97)	*T*2 51–57 (n = 105)	*T*3 ≥57 (n = 87)	*P*-value	*T*1 ≤45 (n = 104)	*T*2 45–51 (n = 99)	*T*3 ≥51 (n = 86)	*P*-value
		Mean ± SD				Mean ± SD				Mean ± SD			
Age (year)	36.60 ± 8.47	35.92 ± 9.23	36.73 ± 8.05	37.31 ± 7.91	0.248^*∗*^	33.81 ± 8.20^a b^	37.75 ± 8.63^a b^	38.32 ± 7.86	0.017^*∗*^	36.96 ± 8.59	36.71 ± 8.35	36.05 ± 8.52	0.264^*∗*^
Physical activity (MET/h/w)	1208.55 ± 2110.75	834.58 ± 746.70	1510.90 ± 2986.93	1366.84 ± 2122.87	0.069	842.78 ± 842.68^b^	969.97 ± 829.24^c^	1926.41 ± 3600.56^b c^	0.001	1426.69 ± 2290.38	1027.28 ± 1236.17	1148.98 ± 2590.20	0.403
Economic status n (%)					0.076				0.104				0.720
Low	67 (100.0)	21 (31.3)	25 (37.3)	21 (31.3)		15 (22.4)	17 (25.4)	35 (52.2)		27 (40.3)	23 (34.3)	17 (25.4)	
Medium	138 (100.0)	55 (39.9)	40 (29.0)	43 (31.2)		50. (36.2)	58 (42.0)	30 (21.7)		45 (32.6)	42 (30.4)	51 (37.0)	
High	71 (100.0)	31 (43.7)	21 (29.6)	19 (26.8)		27 (38.0)	25 (35.2)	19 (26.8)		29 (40.8)	26 (36.6)	16 (22.5)	
Occupation status n (%)					0.206				0.780				0.819
Unemployed	189 (100.0)	58 (30.7)	63 (33.3)	68 (36.0)		72 (38.1)	61 (32.3)	56 (29.6)		67 (35.4)	65 (34.4)	57 (30.2)	
Employed	94 (100.0)	48 (51.1)	27 (28.7)	19 (20.2)		23 (24.5)	40 (42.6)	31 (33.0)		34 (36.2)	32 (34.0)	28 (29.8)	
Marital status n (%)					0.334				0.364				0.817
Single	55 (100.0)	25 (45.5)	12 (21.8)	18 (32.7)		23 (41.8)	20 (36.4)	12 (21.8)		18 (32.7)	15 (27.3)	22 (40.0)	
Married	224 (100.0)	85 (37.9)	75 (33.5)	64 (28.6)		70 (31.3)	84 (37.5)	70 (31.3)		82 (36.6)	79 (35.3)	63 (28.1)	
Education status n (%)					0.213				0.774				0.125
Low	43 (100.0)	10 (23.3)	13 (30.2)	20 (46.5)		10 (23.3)	12 (27.9)	21 (48.8)		10 (23.3)	16 (37.2)	17 (39.5)	
Diploma	86 (100.0)	29 (33.7)	31 (36.0)	26 (30.2)		30 (34.9)	31 (36.0)	25 (29.1)		29 (33.7)	32 (37.2)	25 (29.1)	
High	159 (100.0)	72 (45.3)	46 (28.9)	41 (25.8)		56 (35.2)	62 (39.0)	41 (25.8)		65 (40.9)	50 (31.4)	44 (27.7)	
Supplement use n (%)					0.318				0.735				0.036
Yes	150 (100.0)	61 (40.7)	48 (32.0)	41 (27.3)		49 (32.7)	53 (35.3)	48 (32.0)		62 (41.3)	48 (32.0)	40 (26.7)	
No	115 (100.0)	39 (33.9)	38 (33.0)	38 (33.0)		40 (34.8)	44 (38.3)	31 (27.08)		33 (28.7)	44 (38.3)	38 (33.0)	
Anthropometric measurements													
Weight (kg)	80.70 ± 12.26	79.5 ± 11.01	80.90 ± 11.96	81.94 ± 13.98	0.967	81.43 ± 13.49	80.44 ± 11.59	80.19 ± 11.71	0.203	79.81 ± 11.68	83.52 ± 13.57	78.53 ± 10.81	0.123
Height (cm)	161.25 ± 5.93	160.74 ± 5.98	162.24 ± 6.36	160.86 ± 5.32	0.048	162.25 ± 6.08	160.93 ± 5.57	160.50 ± 6.10	0.875	161.54 ± 5.40	160.69 ± 5.85	161.54 ± 6.62	0.630
BMI (kg/m^2^)	31.06 ± 4.33	30.78 ± 4.21	30.68 ± 3.98	31.79 ± 4.76	0.545	30.92 ± 4.84	31.08 ± 4.13	31.18 ± 3.99	0.224	30.58 ± 4.17^a^	32.31 ± 4.59^a c^	30.20 ± 3.91^c^	0.020
WHR	0.93 ± 0.05	0.92 ± 0.05	0.93 ± 0.05	0.93 ± 0.04	0.507	0.93 ± 0.05	0.92 ± 0.05	0.92 ± 0.05	0.862	0.93 ± 0.05^a^	0.94 ± 0.05^a^	0.92 ± 0.05	0.027
Body fat (%)	41.53 ± 5.55	41.15 ± 5.68	41.17 ± 4.83	42.35 ± 6.03	0.102	41.31 ± 5.83	41.32 ± 5.43	42.01 ± 5.40	0.242	40.70 ± 5.86^a^	43.12 ± 5.50^a^	40.72 ± 4.85	0.024
Fat free mass (kg)	46.76 ± 5.59	46.34 ± 5.31	47.17 ± 5.53	46.86 ± 6.01	0.163	47.06 ± 5.50	46.89 ± 5.39	46.26 ± 5.95	0.092	46.73 ± 5.37	47.12 ± 5.87	46.38 ± 5.56	0.852
Visceral fat area (CM^2^)	163.18 ± 38.69	158.74 ± 38.60	162.14 ± 37.38	169.86 ± 39.66	0.209	164.63 ± 40.18	161.23 ± 38.41	163.87 ± 37.68	0.448	159.27 ± 39.28^a^	173.89 ± 40.28^a^	155.61 ± 33.48	0.010
Inflammatory markers													
TGF-*β*1 (pg/ml)	29088.18 ± 6167.49	29583.08 ± 6296.11	28618.99 ± 6549.15	28863.60 ± 5661.96	0.615	30473.07 ± 5648.48	28988.70 ± 6184.76	28005.47 ± 6437.75	0.308	28487.44 ± 6058.51	29635.33 ± 6370.66^c^	29287.33 ± 6141.14^c^	0.270
IL-*β*1 (pg/ml)	2.72 ± 0.93	2.58 ± 0.91	3.16 ± 0.96	2.60 ± 0.88	0.397	2.74 ± 0.69	2.72 ± 1.03	2.71 ± 0.99	0.570	2.76 ± 1.06	2.67 ± 0.93	2.75 ± 0.85	0.540
MCP1 (pg/ml)	50.88 ± 92.82	52.62 ± 87.35	53.17 ± 105.17	46.11 ± 87.75	0.676	52.20 ± 63.22	53.68 ± 108.82	46.21 ± 96.56	0.986	51.37 ± 77.61	44.92 ± 90.61	57.15 ± 110.85	0.622
Hs-CRP (pg/ml)	4.24 ± 4.59	4.22 ± 4.96 b	4.18 ± 4.73	4.32 ± 3.97^b^	0.025	4.27 ± 4.41	4.39 ± 4.75	4.06 ± 4.62	0.324	3.65 ± 4.04	4.99 ± 4.82	4.12 ± 4.91	0.086
Clinical measurements													
SBP (mm/Hg)	111.63 ± 13.79	110.11 ± 12.79	110.86 ± 11.73	114.36 ± 16.50	0.872	111.58 ± 14.25	109.79 ± 13.56	113.92 ± 13.36	0.084	111.08 ± 13.04	112.15 ± 15.54	111.69 ± 12.68	0.102
DBP (mm/Hg)	77.73 ± 9.64	77.66 ± 8.63	76.14 ± 8.92	79.47 ± 11.26	0.820	77.51 ± 9.82	77.09 ± 8.71	78.77 ± 10.50	0.510	77.10 ± 9.31 a	77.60 ± 10.70	78.65 ± 8.77^a^	0.002
Biochemistry assessments													
TG (mmol/L)	1.38 ± 0.79	1.41 ± 0.80	1.24 ± 0.58	1.467 ± 0.93	0.626	1.39 ± 0.73	1.29 ± 0.79	1.46 ± 0.84	0.698	1.31 ± 0.75	1.43 ± 0.83	1.39 ± 0.79	0.008
LDL-C (mmol/L)	2.45 ± 0.62	2.46 ± 0.65	2.44 ± 0.60	2.43 ± 0.61	0.584	2.35 ± 0.62	2.48 ± 0.64	2.50 ± 0.60	0.920	2.47 ± 0.64 b	2.50 ± 0.63	2.35 ± 0.59 b	0.029
HDL-C (mmol/L)	1.20 ± 0.28	1.21 ± 0.28	1.22 ± 0.28	1.17 ± 0.28	0.812	1.19 ± 0.25	1.22 ± 0.29	1.20 ± 0.28	0.984	1.24 ± 0.29	1.195 ± 0.25	1.17 ± 0.28	0.625
Karelis (score)	2.65 ± 1.21	2.59 ± 1.27	2.79 ± 1.13	2.59 ± 1.22	0.503	2.52 ± 1.20	2.78 ± 1.27	2.62 ± 1.16	0.408	2.83 ± 1.04	2.38 ± 1.32	2.73 ± 1.25	0.043

Abbreviations: BMI, body mass index; DBP, diastolic blood pressure; FBS, fasting blood sugar; HDL, high-density lipoprotein; hPDI, healthful plant-based diet index; IL-*β*1, interleukin-beta 1; LDL, low-density lipoprotein; MCP1, monocyte chemoattractant protein-1; PDI, overall plant-based diet index; SBP, systolic blood pressure; TGF-*β*1, transforming growth factor beta 1; WHR, waist-hip ratio; uPDI, unhealthful plant-based diet index, Values are mean ± standard deviation (SD) for continuous variables and percentage for dichotomous variables. Using one-way ANOVA for continuous variables and Chi-square test for categorical variables. ^*∗*^*P*-value adjusted for age, BMI, physical activity, energy intake. *P*-value <0.05 significant. ^a^Significant mean difference between tertiles one and two. ^b^Significant mean difference between tertiles one and three. ^c^Significant mean difference between tertiles two and three.

**Table 2:** Dietary intake of participants across the tertiles of PDI, hPDI, and uPDI scores in overweight and obese women (*n* = 289).

	Total (n = 289)	PDI				hPDI				uPDI			
		*T*1 ≤51 (n = 111)	*T*2 51–57 (n = 90)	*T*3 ≥57 (n = 88)	*P*-value	*T*1 ≤51 (n = 97)	*T*2 51–57 (n = 105)	*T*3 ≥57 (n = 87)	*P*-value	*T*1 ≤45 (n = 104)	*T*2 45–51 (n = 99)	T3 ≥51(n = 86)	*P*-value
		Mean ± SD				Mean ± SD				Mean ± SD			
Energy intake (kcal/d)	2617.15 ± 752.65	2199.67 ± 596.97	2665.23 ± 721.25	3094.57 ± 661.22	<0.001	3023.98 ± 702.77	2438.72 ± 650.17	2378.92 ± 742.69	<0.001	2858.48 ± 724.48	2570.01 ± 739.70	2379.58 ± 722.21	<0.001
Macronutrients													
Protein (g/d)	88.43 ± 28.50	80.35 ± 26.36	90.49 ± 31.42	96.53 ± 25.43	<0.001^*∗*^	102.50 ± 29.14	83.75 ± 25.45	78.40 ± 25.24	0.146^*∗*^	104.44 ± 30.26	85.42 ± 21.49	72.55 ± 23.05	<0.001^*∗*^
Carbohydrate (g/d)	372.59 ± 120.40	302.77 ± 89.16	374.57 ± 104.66	458.64 ± 114.11	<0.001	431.51 ± 109.34	342.68 ± 108.84	343.01 ± 122.41	0.373	396.94 ± 115.63	365.57 ± 123.71	351.23 ± 118.44	0.001
Total fat (g/d)	94.47 ± 33.38	80.39 ± 27.04	98.24 ± 34.16	108.37 ± 33.28	0.166	107.60 ± 31.39	89.47 ± 31.16	85.85 ± 34.01	0.616	104.66 ± 30.52	93.57 ± 33.18	83.17 ± 33.47	0.254
Micronutrients													
Cholesterol (mg/d)	252.67 ± 104.94	246.74 ± 93.31	270.38 ± 124.51	242.04 ± 95.42	<0.001	309.83 ± 117.72	237.50 ± 82.75	207.25 ± 84.13	<0.001	304.25 ± 119.36	241.96 ± 84.96	32.27 ± 12.02	<0.001
SFA (g/d)	28.02 ± 11.13	24.96 ± 9.47	29.67 ± 12.49	30.19 ± 10.84	0.001	33.95 ± 11.99	26.05 ± 8.64	23.79 ± 10.07	<0.001	32.27 ± 12.02	27.02 ± 10.03	24.03 ± 9.042	<0.001
MUFA (g/d)	31.32 ± 11.88	27.01 ± 10.26	32.74 ± 11.76	35.29 ± 12.27	0.300	34.62 ± 10.20	30.18 ± 11.69	29.00 ± 13.09	0.326	34.96 ± 10.58	31.35 ± 12.48	26.87 ± 11.23	0.037
PUFA (g/d)	20.05 ± 9.12	16.15 ± 6.94	20.78 ± 9.03	24.23 ± 9.68	0.042	21.04 ± 7.80	19.51 ± 9.31	19.61 ± 10.21	0.020	21.11 ± 7.39	20.95 ± 9.96	17.74 ± 9.69	0.154
Linoleic acid (g/d)	17.33 ± 8.65	13.70 ± 6.60	18.09 ± 8.66	21.15 ± 9.17	0.022	18.13 ± 7.37	16.84 ± 9.00	17.04 ± 9.55	0.037	17.69 ± 7.09	18.42 ± 9.53	15.65 ± 9.15	0.080
Linolenic acid (g/d)	1.23 ± 0.67	0.96 ± 0.46	1.25 ± 0.67	1.54 ± 0.76	0.047	1.27 ± 0.60	1.17 ± 0.63	1.25 ± 0.79	0.003	1.42 ± 0.61	1.19 ± 0.68	1.03 ± 0.67	0.094
Trans fatty acid (g/d)	0.00 ± 0.00	0.00 ± 0.00	0.00 ± 0.00	0.00 ± 0.00	0.601	0.00 ± 0.00	0.00 ± 0.00	0.00 ± 0.00	0.925	0.00 ± 0.00	0.00 ± 0.00	0.00 ± 0.00	0.826
Na (mg/d)	4244.77 ± 1426.27	3799.71 ± 1275.33	4257.58 ± 1429.39	4793.04 ± 1424.97	0.611	4764.82 ± 1558.12	4109.15 ± 1289.04	3828.62 ± 1261.04	0.407	4446.88 ± 1338.19	4276.88 ± 1211.11	3963.39 ± 1702.97	0.435
K (mg/d)	4320.85 ± 1548.07	3567.15 ± 1296.01	4439.34 ± 1553.59	5150.35 ± 1380.30	0.260	4687.06 ± 1514.02	4023.23 ± 1412.00	4271.75 ± 1671.48	<0.001	5119.67 ± 1469.27	4174.72 ± 1453.25	3523.05 ± 1272.61	<0.001
Fe (mg/d)	18.62 ± 5.94	15.51 ± 4.85	18.91 ± 5.62	22.24 ± 5.40	0.284	20.89 ± 5.60	17.40 ± 5.64	17.57 ± 5.99	0.019	20.24 ± 5.86	18.34 ± 5.68	16.98 ± 5.89	0.965
Calcium (mg/d)	1162.04 ± 414.20	1053.36 ± 366.21	1222.07 ± 474.59	1237.73 ± 378.62	<0.001	1324.22 ± 399.800	1097.17 ± 378.66	1059.50 ± 421.04	0.885	1356.74 ± 435.15	1138.82 ± 353.69	953.32 ± 341.34	<0.001
Mg (mg/d)	457.76 ± 146.97	380.76 ± 118.76	471.36 ± 147.30	540.99 ± 129.28	0.176	502.10 ± 137.84	429.99 ± 140.74	441.85 ± 154.12	0.001	512.39 ± 129.00	453.79 ± 148.28	396.28 ± 142.04	0.003
Zn (mg/d)	12.88 ± 4.19	11.14 ± 3.47	13.33 ± 4.53	14.62 ± 3.82	0.017	14.89 ± 3.91	11.94 ± 3.73	11.77 ± 4.23	0.818	14.65 ± 3.98	12.71 ± 4.00	10.93 ± 3.74	<0.001
Selenium (mg/d)	119.69 ± 42.62	106.27 ± 36.78	122.01 ± 41.73	134.25 ± 45.53	0.056	141.74 ± 39.71	112.15 ± 41.73	104.21 ± 36.84	0.021	124.69 ± 38.57	121.17 ± 40.47	111.95 ± 48.70	0.073
Vitamin A (RAE/d)	772.14 ± 407.42	666.11 ± 320.70	822.60 ± 513.13	854.27 ± 355.38	0.459	821.84 ± 400.33	734.83 ± 471.52	761.75 ± 401.96	0.201	985.33 ± 474.43	712.33 ± 306.92	583.17 ± 291.81	<0.001
*β* carotene	5236.96 ± 3511.29	4254.02 ± 2737.68	5355.38 ± 4221.70	6355.68 ± 3254.51	0.109	4839.20 ± 2665.53	5243.34 ± 3949.80	5672.73 ± 3757.70	<0.001	6692.32 ± 4301.70	4898.24 ± 2646.88	3866.91 ± 2578.58	<0.001
Vitamin C (mg)	195.72 ± 125.53	152.72 ± 93.78	184.71 ± 96.65	261.20 ± 157.06	0.009	209.70 ± 111.16	173.63 ± 94.59	206.77 ± 164.97	0.004	227.84 ± 112.42	194.31 ± 147.84	158.48 ± 101.04	0.136
Vitamin D (*μ*g)	1.95 ± 1.61	1.99 ± 1.48	2.22 ± 2.01	1.64 ± 1.23	<0.001	2.43 ± 1.98	1.79 ± 1.26	1.62 ± 1.40	0.121	2.72 ± 1.89	1.86 ± 1.41	1.13 ± 0.87	<0.001
Vitamin E (mg)	17.34 ± 9.29	14.58 ± 8.59	18.26 ± 10.25	19.87 ± 8.25	0.214	17.59 ± 8.80	17.78 ± 9.93	16.52 ± 9.09	0.078	18.04 ± 8.77	18.58 ± 10.21	15.05 ± 8.47	0.100
Thiamin (mg)	2.08 ± 0.65	1.80 ± 0.55	2.09 ± 0.61	2.41 ± 0.65	0.444	2.42 ± 0.58	1.94 ± 0.60	1.86 ± 0.63	0.166	2.16 ± 0.62	2.06 ± 0.62	1.99 ± 0.72	0.001
Vitamin B6 (mg)	2.15 ± 0.70	1.89 ± 0.64	2.16 ± 0.71	2.48 ± 0.64	0.400	2.41 ± 0.70	2.05 ± 0.64	2.00 ± 0.70	0.494	2.51 ± 0.75	2.07 ± 0.56	1.82 ± 0.59	<0.001
Folate (*μ*g/d)	605.20 ± 175.81	505.09 ± 137.45	618.20 ± 164.68	718.18 ± 157.51	0.001	661.82 ± 161.24	574.23 ± 173.19	579.46 ± 180.98	0.037	654.47 ± 167.55	596.72 ± 173.03	555.38 ± 174.99	0.875
Vitamin B12 (*μ*g/d)	4.33 ± 2.39	4.21 ± 1.94	4.74 ± 3.23	4.06 ± 1.80	<0.001	5.48 ± 3.08	3.96 ± 1.77	3.49 ± 1.56	0.002	5.55 ± 2.99	3.93 ± 1.52	3.31 ± 1.69	<0.001
Total fiber (g/d)	45.18 ± 18.80	36.07 ± 14.55	45.42 ± 17.40	56.41 ± 18.94	0.003	48.21 ± 18.53	41.40 ± 16.27	46.35 ± 21.23	<0.001	48.10 ± 17.94	45.38 ± 18.58	41.40 ± 19.60	0.619
Food groups													
Refined grain (g/d)	431.81 ± 220.51	364.14 ± 171.28	424.49 ± 189.31	524.66 ± 269.42	0.448	519.07 ± 223.80	407.36 ± 194.53	364.05 ± 217.24	0.109	412.30 ± 245.99	425.73 ± 190.53	462.42 ± 219.33	<0.001
Whole grain (g/d)	7.61 ± 10.43	4.05 ± 6.14	8.78 ± 10.11	10.90 ± 13.40	<0.001	5.80 ± 10.49	6.99 ± 9.00	10.38 ± 11.47	<0.001	11.12 ± 13.01	7.35 ± 9.51	3.66 ± 5.32	<0.001
Fruits (g/d)	530.85 ± 338.52	399.57 ± 264.21	514.36 ± 306.20	713.30 ± 373.14	0.007	565.72 ± 328.02	480.94 ± 293.61	552.19 ± 393.10	0.004	626.84 ± 340.54	517.46 ± 338.44	430.17 ± 306.51	0.106
Vegetables (g/d)	434.96 ± 263.48	362.36 ± 233.79	441.97 ± 277.93	519.37 ± 260.20	0.176	392.55 ± 238.87	434.16 ± 276.45	483.22 ± 268.32	<0.001	552.71 ± 302.37	409.79 ± 231.36	321.55 ± 178.90	<0.001
Nuts (g/d)	14.42 ± 16.22	8.98 ± 8.02	15.04 ± 18.74	20.66 ± 18.77	0.059	15.18 ± 15.27	12.62 ± 13.94	15.77 ± 19.46	0.020	17.43 ± 15.48	14.47 ± 16.37	10.74 ± 16.34	0.396
Dairy (g/d)	388.43 ± 246.20	372.84 ± 225.04	421.84 ± 265.70	373.94 ± 250.47	<0.001	474.32 ± 284.63	355.87 ± 204.21	331.97 ± 222.01	0.193	507.78 ± 282.98	368.47 ± 204.39	267.08 ± 165.51	<0.001
Legumes (g/d)	52.80 ± 41.34	40.45 ± 36.08	56.44 ± 37.95	64.66 ± 46.78	0.007	44.12 ± 28.82	53.63 ± 49.01	61.49 ± 41.60	<0.001	67.11 ± 45.51	53.46 ± 41.87	34.75 ± 26.07	<0.001
Sea foods (g/d)	11.30 ± 12.10	13.73 ± 12.70	10.81 ± 13.82	8.72 ± 8.44	<0.001	14.00 ± 14.87	11.55 ± 11.59	7.97 ± 7.87	0.025	16.72 ± 15.09	10.79 ± 10.57	5.32 ± 4.41	<0.001
Plant oils (g/d)	16.89 ± 16.07	14.63 ± 13.58	19.16 ± 20.05	17.41 ± 14.06	0.255	16.08 ± 13.77	18.75 ± 15.62	15.54 ± 18.73	0.181	18.15 ± 18.02	18.81 ± 16.38	13.15 ± 12.29	0.061
Animal oils (g/d)	6.57 ± 12.25	6.08 ± 11.09	7.78 ± 13.22	5.94 ± 12.67	0.015	8.32 ± 13.11	6.55 ± 10.97	4.63 ± 12.58	0.629	10.29 ± 17.23	4.62 ± 7.49	4.30 ± 7.61	0.011

Abbreviations: MUFA, mono unsaturated fatty acid; PUFA, poly saturated fatty acid; SFA, saturated fatty acid. Values are mean ± standard deviation (SD) for continuous variables. Using one-way ANOVA for continuous variables and Chi-square test for categorical variables. ^*∗*^*P*-value adjusted for energy intake. *P*-value <0.05 significant.

**Table 3:** Association of inflammatory markers with PDI, hPDI, and uPDI scores in overweight and obese women (*n* = 289).

	PDI	*P*-value	hPDI	*P*-value	uPDI	*P*-value
	*β* 95% CI		*β* 95% CI		*β* 95% CI	
TGF-*β*1						
Model 1	−0.046 (−182.204, 96.348)	0.544	−0.128 (−250.583, 19.523)	0.093	0.110 (−42.275, 271.783)	0.151
Model 2	0.014 (−180.109, 206.647)	0.892	−0.201 (−347.819, −22.570)	0.026	0.121 (−59.211, 316.404)	0.178
Model 3	0.028 (−190.705, 246.097)	0.802	−0.286 (452.996, −85.258)	0.004	0.161 (37.575, 384.440)	0.106
IL-*β*1						
Model 1	0.044 (−0.024, 0.035)	0.710	0.002 (−0.03, 0.03)	0.989	0.015 (−0.035, 0.040)	0.901
Model 2	0.051 (−0.031, 0.043)	0.738	−0.075 (−0.047, 0.07)	0.593	0.066 (−0.030, 0.049)	0.628
Model 3	0.090 (−0.030, 0.052)	0.592	0.036 (−0.036, 0.045)	0.823	−0.044 (−0.049, 0.036)	0.762
MCP1						
Model 1	−0.062 (−2.712, 0.993)	0.361	−0.004 (−1.914, 1.791)	0.948	0.008 (−1.98, 2.241)	0.903
Model 2	−0.127 (−4.258, 0.669)	0.152	−0.094 (−3.351, 0.811)	0.230	0.040 (−1.708, 2.952)	0.599
Model 3	−0.087 (−4.145, 1.580)	0.378	−0.095 (−3.712, 1.038)	0.268	0.115 (0.007, 1.949)	0.059

Abbreviations: CI, confidence interval; IL-*β*1, interleukin-beta 1; MCP1, monocyte chemoattractant protein-1; TGF-*β*1, transforming growth factor beta 1. Linear regression was used. Model 1: crude Model 2: adjusted for age, BMI, physical activity, energy intake. Model 3: adjusted for model 2 further with occupation status, economic status, supplement consumption, income.

**Table 4:** Association of MHO and MUHO phenotypes across the tertiles of PDI, hPDI, and uPDI scores in overweight and obese women (*n* = 289).

	PDI					hPDI					uPDI				
	*T*1 ≤51 (*n* = 111)	*T*2 51–57 (*n* = 90)	*T*3 ≥57 (*n* = 88)	*P*-trend	*P*-value	*T*1 ≤51 (*n* = 97)	*T*2 51–57 (*n* = 105)	*T*3 ≥57 (*n* = 87)	*P*-trend	*P*-value	*T*1 ≤45 (*n* = 104)	*T*2 45–51 (*n* = 99)	*T*3 ≥51 (*n* = 86)	*P*-trend	*P*-value
	(OR 95% CI)					(OR 95% CI)					(OR 95% CI)				
MUHO															
Model 1	1	0.770 (0.391–1.517)	1.020 (0.479–2.168)	0.427	0.654	1	1.290 (0.613–2.717)	0.750 (0.383–1.467)	0.539	0.320	1	1.354 (0.682–2.687)	1.806 (0.876–3.721)	0.429	0.277
Model 2	1	0.907 (0.357–2.304)	1.400 (0.565–3.469)	0.765	0.566	1	1.837 (0.701–4.814)	0.936 (0.424–2.065)	0.040	0.310	1	1.149 (0.499–2.644)	1.478 (0.633–3.451)	0.752	0.659
Model 3	1	0.697 (0.241–2.016)	0.967 (0.354–2.691)	0.475	0.719	1	1.754 (0.628–4.895)	0.955 (0.396–2.305)	0.038	0.430	1	0.820 (0.315–2.132)	1.094 (0.425–2.814)	0.674	0.821

Abbreviation: CI, confidence interval; MUHO, metabolically unhealthy obesity; OR, odds ratio. Reference group: Metabolic healthy obesity Binary logistic regression was used. Model 1: Crude Model 2: adjusted for age, BMI, physical activity, energy intake. Model 3: adjusted for model 2 further with occupation status, economic status, supplement consumption, income.

**Table 5:** Association of MHO and MUHO phenotypes across the tertiles of PDI, hPDI, and uPDI scores mediated by inflammatory markers in overweight and obese women (*n* = 289).

	Inflammatory markers	PDI					hPDI					uPDI				
		*T*1 ≤51 (*n* = 111)	*T*2 51–57 (*n* = 90)	*T*3 ≥57 (*n* = 88)	*P*-trend	*P*-value	*T*1 ≤51 (*n* = 97)	*T*2 51–57 (*n* = 105)	*T*3 ≥57 (*n* = 87)	*P*-trend	*P*-value	*T*1 ≤45 (*n* = 104)	*T*2 45–51 (*n* = 99)	*T*3 ≥51 (*n* = 86)	*P*-trend	*P*-value
		(OR 95%CI)					(OR 95%CI)					(OR 95%CI)				
MUHO	^£^IL-*β*1	1	3.465 (0.348, 34.486)	1.104 (0.137, 8.877)	0.300	0.503	1	8.743 (0.655, 116.769)	0.934 (0.151, 5.775)	0.140	0.174	1	0.828 (0.115, 5.957)	1.759 (0.248, 12.461)	0.866	0.743
	^€^MCP-1	1	0.767 (0.241, 2.434)	1.220 (0.408, 3.646)	0.565	0.631	1	1.438 (0.498, 4.156)	0.881 (0.348, 2.230)	0.517	0.608	1	0.789 (0.288, 2.160)	1.007 (0.377, 2.685)	0.635	0.854
	^¥^TGF-*β*1	1	0.391 (0.101, 1.520)	0.785 (0.214, 2.880)	0.156	0.327	1	1.276 (0.346, 4.701)	0.951 (0.309, 2.932)	0.738	0.894	1	0.565 (0.157, 2.031)	0.749 (0.211, 2.656)	0.380	0.681

Abbreviation: IL*β*-1, interleukin beta-1; MCP1, monocyte chemoattractant protein; MUHO, metabolically unhealthy obesity; TGF*β*-1, transforming growth factor beta-1. Reference group: Metabolic healthy obesity, OR, odds ratio. Binary logistic regression was used. ^£^Adjusted with age, BMI, physical activity, energy intake, occupation status, economic status, supplement consumption, income, and IL-*β*1. ^€^Adjusted with age, BMI, physical activity, energy intake, occupation status, economic status, supplement consumption, income, and MCP-1. ^¥^Adjusted with age, BMI, physical activity, energy intake, occupation status, economic status, supplement consumption, income, and TGF-*β*1.

## Data Availability

It is available if needed.

## Abbreviations

BMI: Body mass index
DBP: Diastolic blood pressure
FBS: Fasting blood sugar
HDL: High density lipoprotein
hPDI: Healthful plant-based diet index
IL-*β*1: Interleukin-beta 1
LDL: Low density lipoprotein
MCP1: Monocyte chemoattractant protein-1
PDI: Overall plant-based diet index
SBP: Systolic blood pressure
TGF-*β*1: Transforming growth factor beta 1
WHR: Waist-hip ratio
uPDI: Unhealthful plant-based diet index
MUFA: Mono unsaturated fatty acid
PUFA: Poly saturated fatty acid
SFA: Saturated fatty acid
CI: Confidence interval
MUHO: Metabolically unhealthy obesity
OR: Odds ratio
MHO: Metabolic healthy obesity
WC: Waist circumference
PBD: Plant-based diet
DASH: Dietary approaches to stop hypertension
CRP: C-reactive protein
WHO: World health organization
HOMA: Homeostatic model assessment
FFQ: Food frequency questionnaire
FCT: Food composition table
USDA: United states department of agriculture
IPAQ: Physical activity questionnaire
MetS: Metabolic syndrome
PPAR: Peroxisome proliferator-activated receptor
FFAs: Free fatty acids.
